# The SmartGlove BISINDO Wearable Sensor and Large Language Model–Based Sign Language–to-Speech System for Users With Communication Disorders: Design Science Research Study

**DOI:** 10.2196/96258

**Published:** 2026-10-22

**Authors:** Suhartono Suhartono, Bambang Yulianto, Burhanuddin Arafah, Kusubakti Andajani, Abdul Kholiq, Muhammad Ridha Anugrah Latief, Bambang Prastio, Diding Wahyudin Rohaedi, Deshinta Arrova Dewi, Taqdir Taqdir

**Affiliations:** 1 Department of Indonesian Language and Literature Education, Universitas Negeri Surabaya, Jalan Lidah Wetan, Lakarsantri, Surabaya, East Java, 60213, Indonesia, +62 81330721085; 2 Hasanuddin University, Makassar, South Sulawesi, Indonesia; 3 State University of Malang, Malang, East Java, Indonesia; 4 INTI International University, Putra Nilai, Negeri Sembilan, Malaysia

**Keywords:** assistive communication technology, sign language recognition, natural language generation, large language model, wearable sensing system, BISINDO, communication disorders, augmentative and alternative communication, inclusive technology, process innovation, Bahasa Isyarat Indonesia

## Abstract

**Background:**

In Indonesia, people with communication disorders predominantly communicate using Bahasa Isyarat Indonesia (BISINDO). Nevertheless, all current systems for BISINDO recognition generate fragmented, word-by-word output and lack natural language generation (NLG) capabilities, thereby fundamentally constraining their communicative utility.

**Objective:**

The aim of this study is to develop, validate, and field-evaluate SmartGlove BISINDO, an AI-assisted wearable sign language–to-speech system that embeds large language model (LLM)–powered NLG into a BISINDO translation pipeline for the first time.

**Methods:**

This study followed the design science research (DSR) framework, covering phases 1 through 5 (problem identification, solution objectives, prototype design and development, demonstration, and evaluation). Data were collected from 12 purposively selected informants (6 domain experts and 6 deaf education companions/teachers) across 3 Indonesian cities (Malang, Surabaya, and Makassar), using a needs-analysis questionnaire and an expert validation sheet, and analyzed through statistical triangulation of agreement percentage, content validity ratio (CVR), and Aiken V. The validated prototype was field-tested in Makassar and Malang with 15 participants with communication disorders and 4 observers or companions; it was evaluated by a disability expert in Surabaya.

**Results:**

The needs analysis yielded a grand mean of 4.77 (SD 0.51) across 4 clusters, with 89% mean agreement, indicating very high priority for all identified needs. The prototype’s 5-layer architecture integrates wearable sensors, on-device gesture classification, and LLM-based NLG. Expert validation showed an overall Aiken V of 0.94, with all 14 items exceeding the 0.75 validity cutoff and with 11/14 items (79%) rated highly valid (V>0.90). The grand mean CVR was 0.76 (SD 0.16), exceeding the Lawshe minimum threshold of 0.56 (*P*<.05), with 3 items—gesture differentiation, AI contextual relevance, and settings configuration ease—falling below the threshold and indicating areas for refinement. During the testing phase, the system received a lower practicality score (mean 3.35, SD 0.16; 67%), particularly for clarity of speech output. However, observers and companions gave a significantly higher score of 88%, with a mean of 4.40 (SD 0.08). A disability expert also gave a score of 81% (with a mean value calculated across the 20 questionnaire items of 4.05, SD 0.60). Regarding language, the AI-generated output underwent three transformation processes: addition, deletion, and substitution. Of the 3, substitution is the most likely to alter the intended meaning.

**Conclusions:**

SmartGlove BISINDO is a wearable sign language–to-speech system that introduces the BISINDO research landscape to LLM-based NLG connect-and-translate approaches and demonstrates strong content validity. This study presents preliminary evidence toward a replicable model for interdisciplinary DSR-based prototype development that combines augmentative and alternative communication (AAC) theory, systemic functional linguistics, and design science methodology, intended as a reference for other underrepresented sign language contexts in Southeast Asia and beyond. Field evaluation further indicated favorable practicality overall, alongside a gap between expert-perceived feasibility and user-rated experience that will guide subsequent design refinement.

## Introduction

### Background

Communication is a fundamental human capacity that enables individuals to express thoughts and needs while building the social relationships necessary for daily life. Around 1.3 billion people worldwide have some form of disability [1]; despite being described as a “global crisis,” communication disorders remain underrepresented in global health policy, even though their negative impacts on education, employment, and social participation, as well as their associated psychosocial effects such as stigma, low self-confidence, and reduced quality of life, are well documented [2-4]. Effective assistive systems for this population must therefore be natural, meaningful, and socially appropriate [2,3]. In Indonesia, approximately 1.02% of the population experiences severe communication difficulties [5], resulting in systematic marginalization for hundreds of thousands of people.

Most deaf people in Indonesia communicate using Indonesian Sign Language (Bahasa Isyarat Indonesia [BISINDO]), which emerged naturally within the Indonesian deaf community and has its own grammar and vocabulary distinct from spoken Indonesian [6]. Because very few people outside the deaf community understand BISINDO, individuals who are deaf face limited opportunities for social interaction [7]. This study responds to the growing need for pragmatic assistive technology (AT) research addressing unmet rehabilitation needs [8], building on recent evidence confirming the feasibility and value of novel AT solutions for this population [9].

### Current Trends and Challenges in Sign Language Recognition Technology

Several previous studies on BISINDO recognition have shown a consistent relationship between accuracy, sentence processing capability, and practical application. For example, a convolutional neural network–long short-term memory (CNN-LSTM) architecture achieved individual gesture classification accuracy of up to 96%, but only for isolated word-level gestures, not for continuous sentences [10]. A mobile CNN-MobileNetV2-based app enabled real-time BISINDO-to-speech conversion via camera, but its classification accuracy was only 55% across 30 gesture categories. This significant decrease limits its reliability in the real world [11]. More recent research has attempted to extend recognition to the sentence level using 3D CNN-LSTM and 3D CNN–bidirectional LSTM (CNN-BiLSTM) models with connectionist temporal classification. These studies successfully overcome the limitations of isolated gestures, but these approaches remain camera-based and only go as far as mapping gestures to text, without natural language generation (NLG) capabilities [12]. Related research developments also come from the 2026 SIBI (Indonesian Sign System) recognition system, which integrates LSTM-based gesture recognition with Indo Bidirectional Encoder Representations from Transformers (BERT) contextual modeling. Word prediction accuracy reached 89%, but that was only for word-level correction, not full sentence generation. The system also did not specifically address BISINDO [13]. On the other hand, a 2025 transformer-based benchmark using the Sign Pose-based Transformer (SPOTER), Siformer, and Multiscale Vision Transformer version 2 (MViTv2) architectures achieved up to 97% accuracy in isolated gesture classification. Meanwhile, a 2026 comparison between MobileNetV3 and EfficientNetV2B0 successfully improved the device’s efficiency. However, both were still limited to gesture classification, without addressing sentence-level meaning [14,15]. Across these studies, 3 limitations recurred: reliance on camera input (rather than wearable sensing), focus on isolated word or gesture recognition, and lack of generative NLG capabilities.

For resource-intensive sign languages, sign language recognition has advanced beyond sentence processing. Transformer-based approaches, for example, are capable of performing end-to-end recognition and translation simultaneously [16]. Sequential parallelism across continuous signage has also been modeled by CNN–hidden Markov model systems that use deep learning on sequential data [17,18]. However, these advances were made in American Sign Language and Deutsche Gebärdensprache, which have largely focused on annotated corpora. BISINDO lacks such resources. Therefore, existing architectures and training methods cannot be readily adapted to sign languages with lower resources and different structures like BISINDO.

Beyond BISINDO-specific work, the broader sign language translation literature has moved decisively toward large language model (LLM)–based, gloss-to-text pipelines conceptually similar to the present system. One approach couples a lightweight Sign Pose-based Transformer (SPOTER) with an off-the-shelf LLM prompted to convert sequences of spotted glosses into fluent spoken-language sentences, requiring no additional translation-specific training or paired corpora [19]. Others have extended this idea to explicitly low-resource sign languages through in-context prompting rather than fine-tuning, to compensate for scarce parallel data [20]. However, a 2025 systematic review of LLM- and multimodal-LLM–based sign language recognition and translation work covering 2020‐2025 cautions that most reported gains remain concentrated in a small number of well-resourced sign languages, chiefly American Sign Language, and that evaluation continues to rely heavily on machine-translation metrics such as Bilingual Evaluation Understudy (BLEU), which are known to be weak proxies for communicative adequacy and are rarely validated with deaf or hard-of-hearing end users [21]. This pattern reinforces the gap motivating the present study: existing gloss/SPOTER-to-LLM pipelines have so far been designed and evaluated for camera-based, high-resource sign languages, and none have been extended to a wearable-sensor pipeline or evaluated directly with the target BISINDO user population.

The current BISINDO system does not incorporate automatic NLG to transform detected gesture sequences into semantically coherent complete sentences. This is a critical gap. Sign languages convey meaning at the level of complete sentences, not just isolated gestures but also using gestures in context. In other words, systems not designed for AAC (augmentative and alternative communication) do not allow users to share the same language as their interlocutors [2,3]. Other researchers have overlooked some of the real potential of AAC from LLMs. For example, an LLM-based interface achieved a 29% text input rate in AAC users with amyotrophic lateral sclerosis [22]. In 2025, another study incorporated an LLM into a social support robot intended to interact with humans in real-time as part of geriatric care. The results showed a significant increase in interaction success and user engagement compared to a non-LLM dialogue system [23]. This demonstrates that LLMs as part of assistive devices for real-time interaction are not only capable but also beneficial in many areas. However, at the level of BISINDO translation, this potential is substantially underused.

### Theoretical Framework

Three theoretical perspectives converge in this research, together informing the design science research (DSR) process’s rigor cycle, each addressing a different layer of the same design problem. AAC theory sets the functional goal: assistive technology (AT) output must be natural and socially acceptable [2,3], a standard that isolated words, carrying no social or contextual meaning, cannot meet. Systemic functional linguistics (SFL) translates this goal into concrete linguistic properties that sign languages, like all languages, express through 3 simultaneous metafunctions [24]: the ideational metafunction conveys experiential content, the interpersonal metafunction encodes the social relationship between speaker and listener, and the textual metafunction structures the message as a coherent whole. Lexical translation alone cannot meet these functional criteria; LLM-based NLG, built on transformer architectures [25], provides the computational mechanism to derive grammatical, coherent, context- and intent-appropriate text from a queue of detected gestures [22,23]. In this chain, AAC theory defines the target, SFL specifies the linguistic properties required to reach it, and LLM-based NLG provides the technical means of fulfilling that demand. This chain directly informed the expert validation instrument, whose items evaluate grammaticality, naturalness, and contextual relevance, operationalizing the SFL metafunctions as validity metrics for the prototype.

### Study Objectives

Within this framework, this study aims to design and evaluate the BISINDO SmartGlove, which will be recognized as a wearable device in the context of AI and the implementation of a sign language–to-speech system based on DSR [26,27]. In this study, DSR phases 1 to 5 were carried out: problem identification and motivation; definition of solution objectives; design and development of artifacts carried out through demonstration and evaluation [28]. This study focuses on indexing the theories of AAC, SFL, and DSR under one interdisciplinary lens. The study hopes that the DSR-based prototype development model can serve to develop inclusive AI AT. This can provide a template for other researchers that can be used in underrepresented sign language environments. Practically, this research meets the communication needs of Indonesian users with severe speech disorders. This is directly related to SDG (Sustainable Development Goal) 4 on quality education and SDG 10 on reducing inequalities. The way to do this is to allow people with hearing impairments and speech disorders to communicate independently in schools and social spaces. SmartGlove BISINDO supports SDG 4, which promotes equal access to education for persons with disabilities, and SDG 10, which calls for full social, economic, and political inclusion regardless of disability.

## Methods

### Research Design and Framework

This study follows 2 complementary DSR frameworks: Hevner [29] 3-cycle model and Peffers et al [27] 6-phase DSR, which covers problem identification, solution objectives, design and development, demonstration, evaluation, and communication. The DSR is one of the most widely adopted procedural frameworks in DSR [28]. DSR was chosen because it is specifically tailored to research that results in innovative technological artifacts as solutions to real-world problems. This iterative, user-centered approach reflects established AT design practices that incorporate stakeholder input throughout development [26-30]. The relevance cycle is operationalized via an analysis of needs with 12 participants, the design cycle through iterative design of a 5-layer prototype, and the rigor cycle through AAC theory and SFL as the guiding knowledge bases, as summarized in Figure 1.

**Figure 1.**
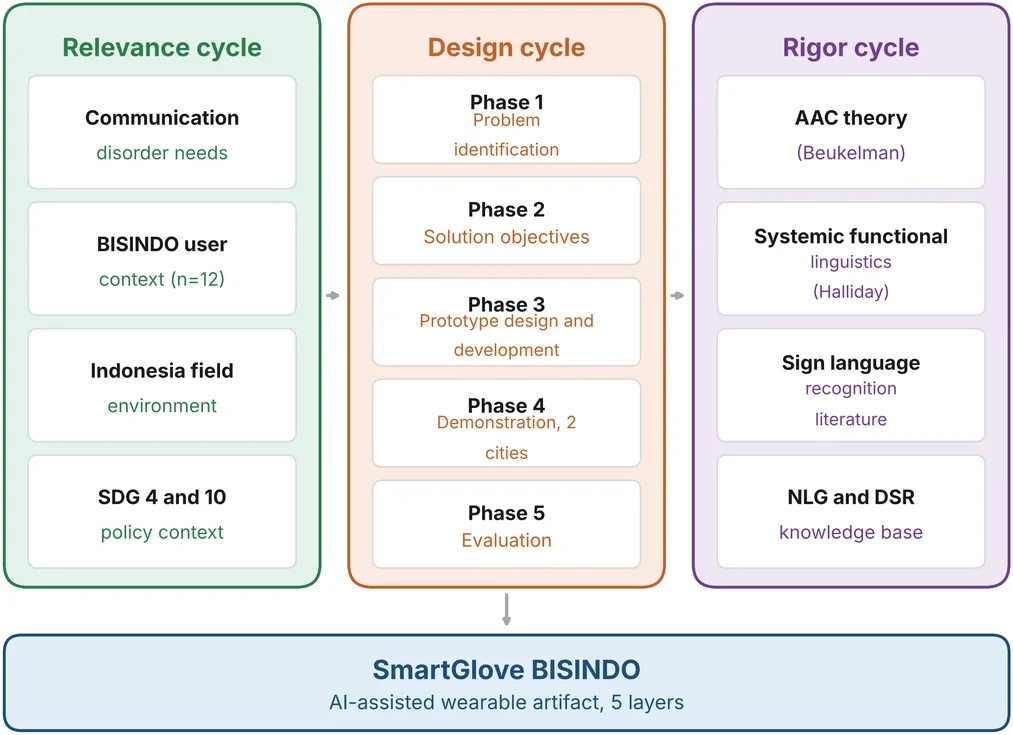
Design science research (DSR) framework of SmartGlove Bahasa Isyarat Indonesia (BISINDO) study [26]. AAC: augmentative and alternative communication; NLG: natural language generation; SDG: Sustainable Development Goal.

### Instrument Development

Both instruments were developed specifically for this study rather than adapted from preexisting validated scales, reflecting the absence of standardized measurement tools for BISINDO-specific, LLM-integrated AT. Item development followed a theory-driven operationalization process: constructs were first derived from the 3 theoretical perspectives described above (AAC theory, SFL, and DSR), then translated into observable, measurable indicators. For example, the SFL textual and interpersonal metafunctions were operationalized into needs-analysis items on sentence completeness and context-appropriate register and into validation items on grammaticality, naturalness, and contextual relevance. Both instruments operationalize the theoretical constructs associated with the DSR relevance and rigor cycles [27]. Beyond this theory-driven design process, construct validity was assessed through content validity review by 2 independent experts prior to field use and further evidenced empirically through the content validity ratio (CVR) and Aiken V results reported for each item.

### Patient and Public Involvement

This study involved 6 deaf education companions and teachers from 3 Indonesian cities (Malang, Surabaya, and Makassar) as respondents in the needs analysis and as expert validators. Dataset curation was performed by a certified BISINDO dataset specialist. This informed the key design decisions. After validation, the prototype directly involves users with communication disorders, aligning with JMIR rehab AT’s interest in research that includes relevant knowledge users [8].

Twelve participants were purposively sampled for their expertise and direct experience in communication contexts involving users with communication disorders in Indonesia. Although a sample of 12 is not statistically significant when using the proper computation of CVR, it is sufficient for CVR analysis, as per Warner’s recommendations, since 12 participants meet the minimum required ceiling threshold (CVRthres) of 0.56 at *P*<.05. The participants were divided into 2 groups, namely, 6 domain experts (consisting of 2 sign language specialists, 2 AI technology experts, and 2 linguists) and 6 deaf education companions and teachers drawn from 3 Indonesian cities: Malang (n=2), Surabaya (n=2), and Makassar (n=2). All participants acted as both respondents for needs analysis and validators of prototype quality assessment.

### Needs Analysis

The needs analysis instrument was a questionnaire covering 4 clusters and 10 items, rated on a 5-point Likert scale (full item list in Multimedia Appendix 1), developed to operationalize the theoretical requirements of AAC and SFL within the DSR relevance cycle. Content validity of the questionnaire was reviewed by 2 independent experts, who were not part of the 12-member study sample, before field use. After a brief introduction to the system concept, 12 participants completed a requirements analysis questionnaire. Responses were then analyzed using descriptive statistics, including percentages, means, and SDs. Priority levels were divided into 2 categories: very high (mean ≥4.60) or high (mean 4.00‐4.59).

### Prototype Design

The prototype development was based on the DSR design cycle. Based on requirement analysis and literature review on existing hardware and software, the initial version was further refined based on observations from wearable use in practice and developments in NLG systems. The design resulted in an architecture specification with technical descriptive analysis and system architecture diagrams, which are 5-layered.

### BISINDO Dataset

The BISINDO dataset has been constructed with the help of a real-life handwritten BISINDO gesture specialist. The expert acted as a demonstrator and authenticity validator during curation. Expert reviews were performed to refine all input gestures derived from the BISINDO standard vocabulary reference. To decrease the ambiguity of classification, some gestures were infrequently used or simply too similar to others (like most coordinate conjunctions, certain particular prepositions like “from” and “for,” or some physical verbs like “input” and “extract”). Additionally, the expert enriched the vocabulary with local references. This dataset comprises terms from the social and religious life in Indonesia such as greetings, emergency phrases, and words for extended families. An expert guiding such a process fits in well with user-centered AAC design methods [2,3] and aligns directly with the cultural and linguistic relevance criteria uncovered in needs analysis.

### Expert Validation

The expert validation instrument was designed in a spreadsheet format. It consisted of 3 components and 14 items (full item list in Multimedia Appendix 2), all rated on a 5-point Likert-type scale, designed to operationalize the theoretical constructs underlying the DSR rigor cycle. Prior to the application of the instrument, 2 independent experts evaluated its content validity. Note that these experts are not part of the 12-person sample, who were followed by the research. The 12 participants undertook the role of validators after a complete demonstration of the system architecture. The team used statistical triangulation to validate the data. They examined 3 indices in parallel: percentage agreement, CVR, and Aiken V (where CVR is calculated using the Lawshe formula [31], representing a minimal threshold of 0.56, for n=12 at *P*<.05). In Aiken V [32], items are considered valid if V is greater than or equal to 0.75 and highly valid if V is greater than 0.90. The triangulation of multiple indices is more trustworthy than relying on a single index [30]. This method helps to solve the well-known limitation of using a single outcome between-participants evaluation framework in previous studies reviewing rehabilitation technologies [32]. A sample of 12 participants was purposively selected. The sample consisted of 2 sign language specialists, 2 AI technology experts, 2 linguists, and 6 deaf education companions and teachers drawn from 3 Indonesian cities: Malang, Surabaya, and Makassar. This composition was intended to be interdisciplinary, consistent with the relevance and rigor cycle of the DSR framework. Use of percentage agreement, CVR, and Aiken V concurrently was a check on interrater consistency across this heterogeneous panel. For the sample size, formal interrater reliability coefficients were not calculated.

### Field Demonstration and Evaluation

Following expert validation, the validated prototype was field-tested with user participants and observers/companions in 2 Indonesian cities, Makassar and Malang, with an independent disability-expert assessment conducted in Surabaya, corresponding to DSR phases 4 and 5 (demonstration and evaluation). The field evaluation was measured using 3 instruments from different perspectives, consistent with recommended approaches for evaluating the usability of rehabilitation technologies across multiple stakeholder groups [33]. First, a practicality questionnaire was completed by users with communication impairments. Second, a parallel questionnaire was administered to an observer or companion present during use. Third, a validation instrument was administered by a disability expert. Because each instrument measures something different and results are expected to vary, analyses of these 3 sets were conducted separately. Furthermore, the study analyzed linguistic performance by taking each interaction and comparing the raw word sequences of gestures (system input) with the final sentences generated by the AI (system output). This was done qualitatively. The observed changes were linguistic shifts generated through the AI-assisted process.

### Ethical Considerations

The study was carried out in accordance with the Declaration of Helsinki and applicable national guidelines for research involving human participants. All participants provided written informed consent, including details on the study objective, procedures, voluntary participation, data confidentiality, and withdrawal rights. Twelve participants signed informed consent before data collection. Field demonstration and evaluation participants, comprising 15 user participants with communication disorders, 4 observers or companions, and 1 disability expert, separately provided informed consent directly, obtained with the assistance of a sign language interpreter for user participants with communication disorders. There was no collection of personal health information; data from the 12 expert and stakeholder participants related to their professional knowledge and attitudes toward system design and validation, while data from field demonstration and evaluation participants related solely to their experience using the prototype and their perceptions of its practicality. Data collection for all stages of the research (needs analysis, expert validation, and field demonstration) was conducted in accordance with research ethics. Ethical approval was formally granted as an Ethical Exemption (reference number 183/UN38.16/KEP/EC/2026) by the Research Ethics Committee of the Lembaga Penelitian dan Pengabdian kepada Masyarakat (Institute for Research and Community Service) at Universitas Negeri Surabaya, confirming that the study met the 7 World Health Organization ethical standards of 2011, based on the 2016 Council for International Organizations of Medical Sciences guidelines.

## Results

### Needs Analysis

The needs analysis used a 5-point Likert-scale questionnaire completed by 12 participants, covering 4 clusters and 10 indicators as presented in Table 1.

**Table 1.** SmartGlove Bahasa Isyarat Indonesia (BISINDO) user needs analysis results.

Cluster and indicator	Mean agreement, %	Mean (SD)
Response speed and accuracy		
Speech output <1 second after gesture completion	100	4.92 (0.29)
Real-time gesture recognition without delay	100	4.83 (0.39)
Minimization of unintended gesture detection errors	83	4.67 (0.65)
Naturalness of language output		
Output as complete grammatical sentences	92	4.83 (0.39)
Sentences comprehensible to lay conversation partners	83	4.75 (0.62)
Context adjustment for formal/informal situations	75	4.58 (0.79)
Portability and ease of use		
Lightweight portable device across contexts	75	4.67 (0.78)
Independent operation without assistance	92	4.75 (0.45)
Cultural and linguistic relevance		
Based on authentic BISINDO vocabulary	100	4.92 (0.29)
Coverage of at least 150 daily-life gestures	92	4.75 (0.45)
Total mean score	89	4.77 (0.51)

Across all 4 clusters, the needs analysis showed very high and homogeneous agreement (grand mean 4.77, SD 0.51; mean agreement 89%). Cultural and linguistic relevance had the highest cluster mean (4.84, SD 0.37; mean agreement 96%) and lowest SD, indicating strong consensus among experts; its top items, response speed and accuracy (item 1.1) and authentic vocabulary basis (item 4.1), each achieved unanimous agreement (mean 4.92, SD 0.29; 12/12, 100%). The response speed and accuracy cluster averaged 4.81 (SD 0.44; mean agreement 94%), naturalness of language output averaged 4.72 (SD 0.60; mean agreement 83%) with one item (context adjustment for formal/informal situations) falling into the High rather than very high category (mean 4.58, SD 0.79), and portability and ease of use averaged 4.71 (SD 0.62; mean agreement 83%).

Each cluster translated directly into a design principle for the prototype. The response speed and accuracy requirement set a sub-1-second end-to-end latency budget across the architecture. The naturalness of language output requirement made the NLG module a required rather than optional architectural element, driving the layer 4 design. Portability and independent-operation requirements pushed the hardware toward wearable, microcontroller-based systems with wireless communication that do not depend on external infrastructure. Finally, the requirement for authentic BISINDO vocabulary coverage of at least 150 gestures set the minimum dataset size.

### Prototype Design

Informed by the 4 need clusters, SmartGlove BISINDO was designed as a 5-layer integrated system connecting sensor data acquisition to speech output. The prototype design was based on the DSR design cycle.

The workflow is illustrated in Figure 2. Once set up, both gloves (left as slave and right as master) sample sensor data at 100 Hz at matching times. The left glove itself acquires 5 flex values and 6 inertial measurement unit (IMU) values, which are transmitted to the right glove through ESP-NOW. The right glove uses a data aggregator to aggregate the data with sync timestamping, sending an array of 22 Float32 features per frame over BLE 5.0 to the Android app. The data then goes through a 3-step preprocessing pipeline before being classified by the TFLite (Google LLC) model. Gestures with confidence levels above the determined threshold of 85% or higher were piped into the AI language wrapper to convert NLG-generated text from the LLM into normal sentences in Bahasa Indonesia, and then vocalized using the Google text-to-speech (TTS) Engine detailed in Table 2.

**Figure 2.**
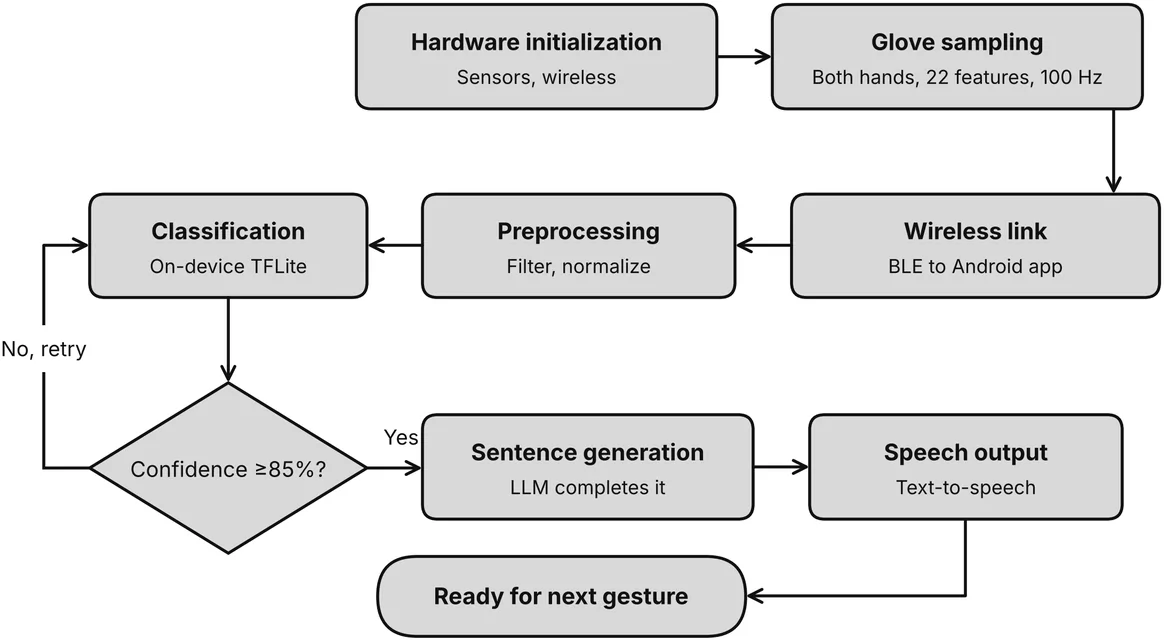
Flowchart of SmartGlove Bahasa Isyarat Indonesia (BISINDO) integrated system architecture. BLE: Bluetooth Low Energy; LLM: large language model.

**Table 2.** Technical specifications: 5-layer SmartGlove Bahasa Isyarat Indonesia (BISINDO) architecture.

Layer	Key components	Key specifications	Primary function
Layer 1: sensor acquisition	ESP32-S3 ZERO (×2), Flex Sensor Spectra 4.5” (×10), MPU^a^-6050 IMU^b^ (×2), and Custom PCB^c^	Dual-core 240 MHz; 12-bit ADC^d^; 100 Hz sampling; 22 features/frame (Float32)	Raw data acquisition from both hands simultaneously
Layer 2: wireless communication	ESP-NOW, BLE^e^ 5.0, and data aggregator	ESP-NOW latency <5 ms; BLE MTU^f^ 512 bytes; throughput 2 Mbps	Sensor data transmission to Android
Layer 3: processing and inference	Preprocessing pipeline, TFLite LSTM^g^+dense, and confidence threshold	Tensor (1,30,22); 150 classes; target accuracy ≥85%; latency <50 ms	On-device BISINDO gesture classification
Layer 4: AI language wrapper	Sentence buffer, LLM^h^ REST^i^ API, and postprocessing UI^j^	Pause-based segmentation; API response max 500 ms; RecyclerView history	LLM-based NLG^k^ for natural Bahasa Indonesia sentence generation
Layer 5: Speech output	Google TTS^l^ engine, Android speaker	Locale id-ID; speed 0.9; real-time playback	Conversion of sentences to Bahasa Indonesia speech

a MPU: motion processing unit.

b IMU: inertial measurement unit.

c PCB: printed circuit board.

d ADC: analog-to-digital converter.

e BLE: Bluetooth Low Energy.

f MTU: maximum transmission unit.

g LSTM: long short-term memory.

h LLM: large language model.

i REST: Representational State Transfer.

j UI: user interface.

k NLG: natural language generation.

l TTS: text-to-speech.

Layer 1 is the physical layer. Five Spectra Symbol 4.5 flex sensors (Spectra Symbol) in each glove measure finger bending, while a single MPU-6050 6-axis IMU (InvenSense/TDK InvenSense) tracks hand orientation, acceleration, and rotation. Combining 2 sensor types yields 22 features per frame. This provides a richer representation than camera-based systems and performance comparable to the validated multi-IMU glove [34]. Dual-core 240-MHz ESP32-S3 (Espressif Systems) ZERO microcontrollers control both gloves at a sampling rate of 100 Hz.

Wireless protocols perform 2 distinct functions at layer 2. The ESP-NOW communicates between the gloves via an inter-ping in less than 5 milliseconds, using peer-to-peer communication, and transmits a Float32 array via BLE 5.0 to the Android application (Google LLC) using a data aggregator, where timestamping is performed as shown in Figure 3.

**Figure 3.**
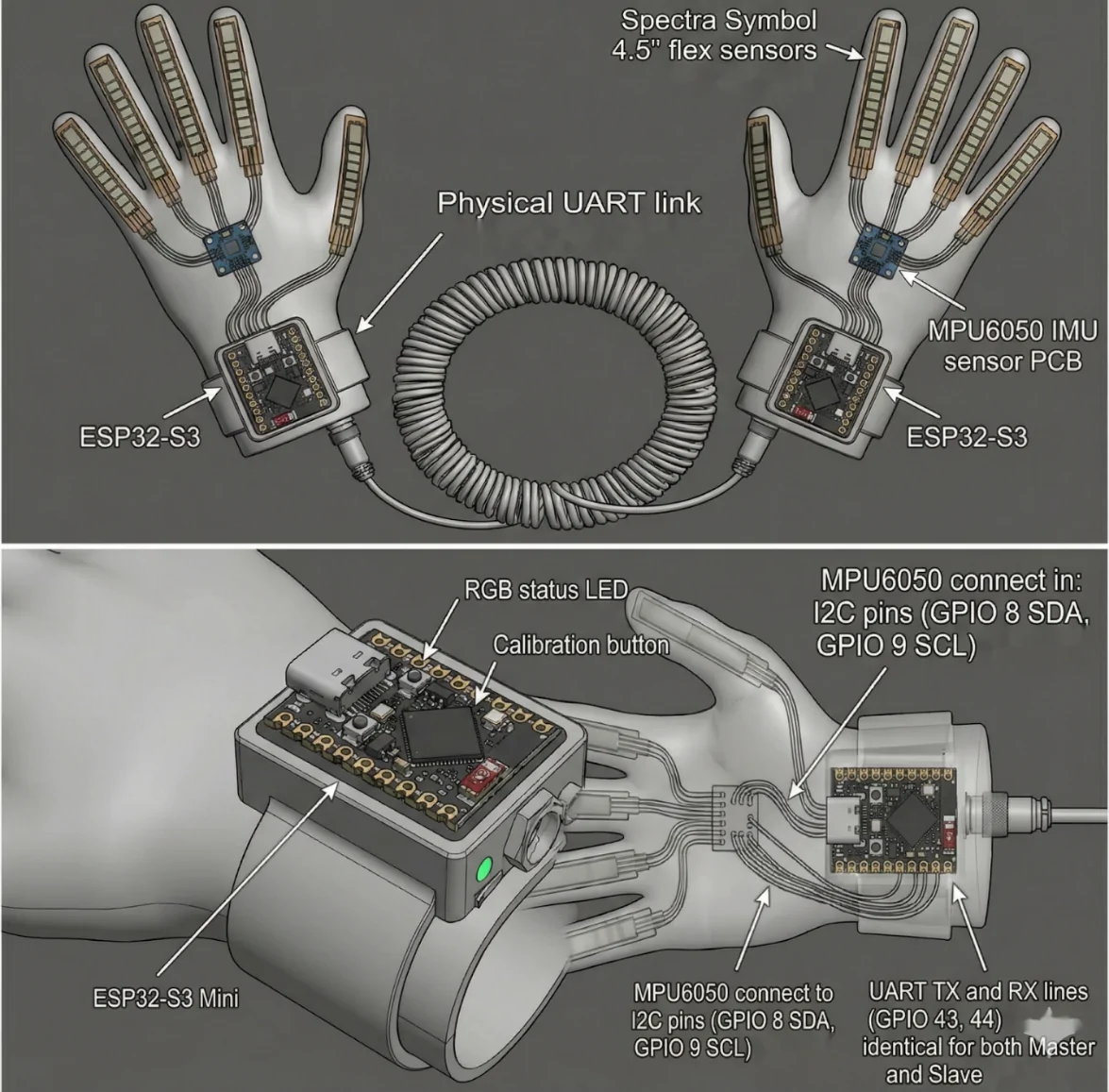
Glove design and specifications. GPIO: general-purpose input/output; I2C: interintegrated circuit; IMU: inertial measurement unit; PCB: printed circuit board; RGB: red-green-blue; RX: receive; SCL: serial clock line; SDA: serial data line; TX: transmit; UART: universal asynchronous receiver-transmitter.

Layer 3 is performed with an input tensor (1, 30, and 22), where the data is passed through a Kalman filter to min-max normalization in a sliding window (30 frames with a stride of 5). The TFLite LSTM+Dense model classifies movements into 150 gestures; only those with 85% or higher accuracy are declared valid. At the same time, on-device inference does not hinder time performance and eliminates cloud reliance, adhering to the portable AT design principles [35].

Layer 4 is the key differentiating component of the BISINDO SmartGlove, integrating LLM-based NLG, which is not present in any previous BISINDO system [36]. Valid gesture labels are collected by the sentence buffer through temporal lag detection. The raw word sequence is sent to the language model via the Representational State Transfer (REST) API (maximum 500 ms) for grammatical and contextual correction, generating sentences like (1) “*Saya sedang makan malam*” from the inputs “*saya*” + “*makan*” + “*malam*”; and (2) “I am having dinner” from the inputs “I” + “eat” + “night.”

The LLM-AAC framework is directly applied to sign language translation. Android’s RecyclerView displays the final sentence in real time and draws data from the local conversation history. At layer 5, the final sentence is converted into Indonesian. The Google TTS Engine is used via locale id-ID with a rate of 0.9. Voice is replayed in real time, and end-to-end latency remains below 1 second throughout the process as shown in Figure 4.

**Figure 4.**
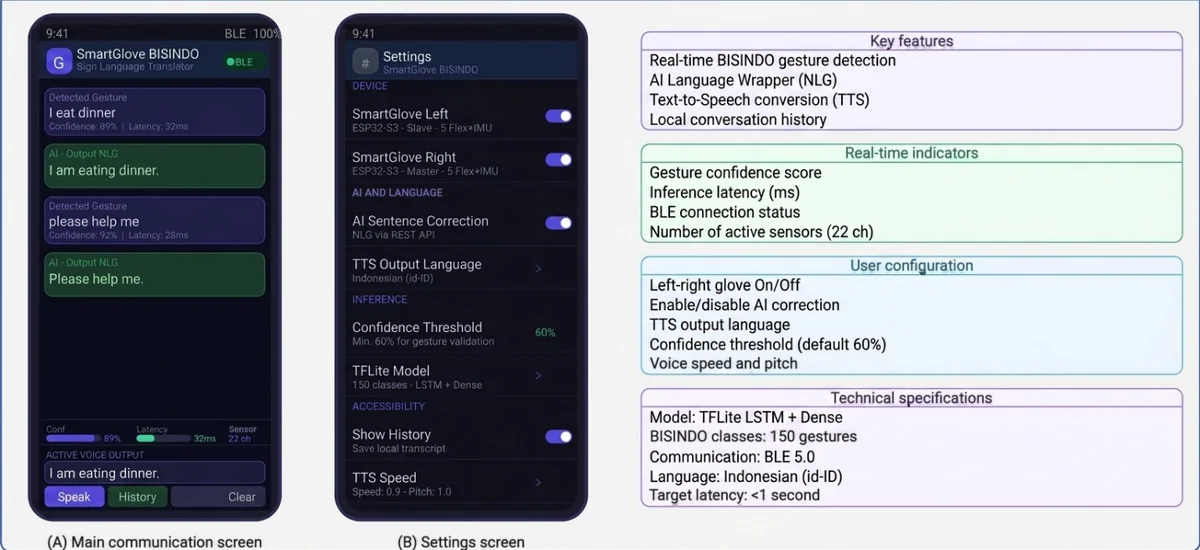
SmartGlove Bahasa Isyarat Indonesia (BISINDO) app interface. BLE: Bluetooth Low Energy; CH: channel; NLG: natural language; LSTM: long short-term memory.

The app interface (Figure 4) is based on 2 primary screens. The communication screen displays the translation step. The raw gestures that are detected, and below them, the sentence corrected by the AI. Quick access to confidence scores, system latency, and BLE connection status indicators, too! Second, there’s a settings screen. You may set up device connections that connect to the inference threshold and voice output update. Accessibility and ease of use (without any specific training) are the whole design around it. This is exactly what the needs analysis showed.

### BISINDO Dataset

The BISINDO gesture dataset comprised 150 classes across 6 categories: 26 letters, 42 numbers, 21 greetings and social expressions, 18 family terms, 2 core essential words, and 41 additional vocabulary items, with 80% (120/150) classes completeness at the time of the field demonstration (full breakdown in Multimedia Appendix 3). This directly addresses the cultural and linguistic relevance criteria identified in the needs analysis and reflects the participatory curation process described in the Methods section.

### Expert Validation

Performance and validity of the prototype were checked using an expert validation sheet. It has 3 areas of interest: greeting the data to calculate how well BISINDO gestures were displayed, accuracy and naturalness should be calculated by NLG-generated sentences, and an app should have a user-friendly interface. Twelve validators completed the sheet. Responses were analyzed using CVR, Aiken V, and triangulated percentage agreement. The summary findings of the study are shown in Table 3.

**Table 3.** Expert validation results: SmartGlove Bahasa Isyarat Indonesia (BISINDO) prototype (N=12).

Code	Assessment item	Values, n (%)	Values, mean (SD)	CVR^a^	Aiken V	Category
K1	BISINDO gesture vocabulary is authentic and meets standards	12 (100)	4.92 (0.29)	1.00	0.98	Highly valid
K2	Coverage of 150 classes represents daily communication needs	11 (92)	4.75 (0.45)	0.83	0.94	Highly valid
K3	The system can differentiate gestures with similar finger configurations	10 (83)	4.67 (0.65)	0.67	0.83	Valid
K4	A flex and IMU^b^ sensor combination is appropriate for capturing BISINDO gesture characteristics	11 (92)	4.83 (0.39)	0.83	0.96	Highly valid
N1	AI-generated sentences are grammatical and conform to Bahasa Indonesia rules	11 (92)	4.83 (0.39)	0.83	0.96	Highly valid
N2	Sentence output sounds natural and communicative to lay conversation partners	10 (83)	4.75 (0.62)	0.67	0.94	Highly valid
N3	AI can correct gesture translation word order into complete sentences	11 (92)	4.83 (0.39)	0.83	0.96	Highly valid
N4	Sentence output is contextually relevant to user gesture intent	9 (75)	4.58 (0.79)	0.50	0.90	Valid
N5	AI response time does not disrupt communication fluency	10 (83)	4.67 (0.65)	0.67	0.92	Highly valid
U1	The interface is easy to understand and operate without specialized training	11 (92)	4.75 (0.45)	0.83	0.94	Highly valid
U2	BLE^c^ connection status and confidence score indicators are clearly presented	10 (83)	4.67 (0.65)	0.67	0.92	Highly valid
U3	Conversation history display helps users monitor communication flow	11 (92)	4.83 (0.39)	0.83	0.96	Highly valid
U4	Settings features (threshold, TTS^d^ speed, language) can be configured easily	9 (75)	4.58 (0.79)	0.50	0.90	Valid
U5	Interface design is inclusive and meets the needs of users with communication disorders	12 (100)	4.92 (0.29)	1.00	0.98	Highly valid
−^e^	Overall mean across all items, n/N (%)	148/168 (88%)	4.76 (0.51)	0.76	0.94	Highly valid

a CVR: content validity ratio.

b IMU: inertial measurement unit.

c BLE: Bluetooth Low Energy.

d TTS: text-to-speech.

e Not applicable.

It performed relatively well on content validity. The average Aiken V was 0.94 (SD 0.04), the mean Likert scale was 4.76 (SD 0.51), and the agreement rate ranged from 88% (148/168) to 100% (12/12) among all technical items tested. No item fell below the threshold of 0.75 for Aiken V, and in fact, 11 out of 14 (79%) items were classified as highly valid (V≥0.90). With an average CVR of 0.76 (SD 0.16) across 12 participants, you can see that this value was well above the Lawshe cutoff number of 0.56. For the separated aspects, aspect 1 received an Aiken V of 0.93, and aspects 2 and 3 had a value of 0.94 each.

However, K3 (overlapping movement), N4 (app context of AI in question), and U4 (low-configurable by hand) were only partly CVRs. The next iteration of the DSR design cycle should have improvements on all three. Figure 5: The Cartesian diagram will clarify this even more. The X-axis is Aiken V; the Y-axis is the mean Likert score.

**Figure 5.**
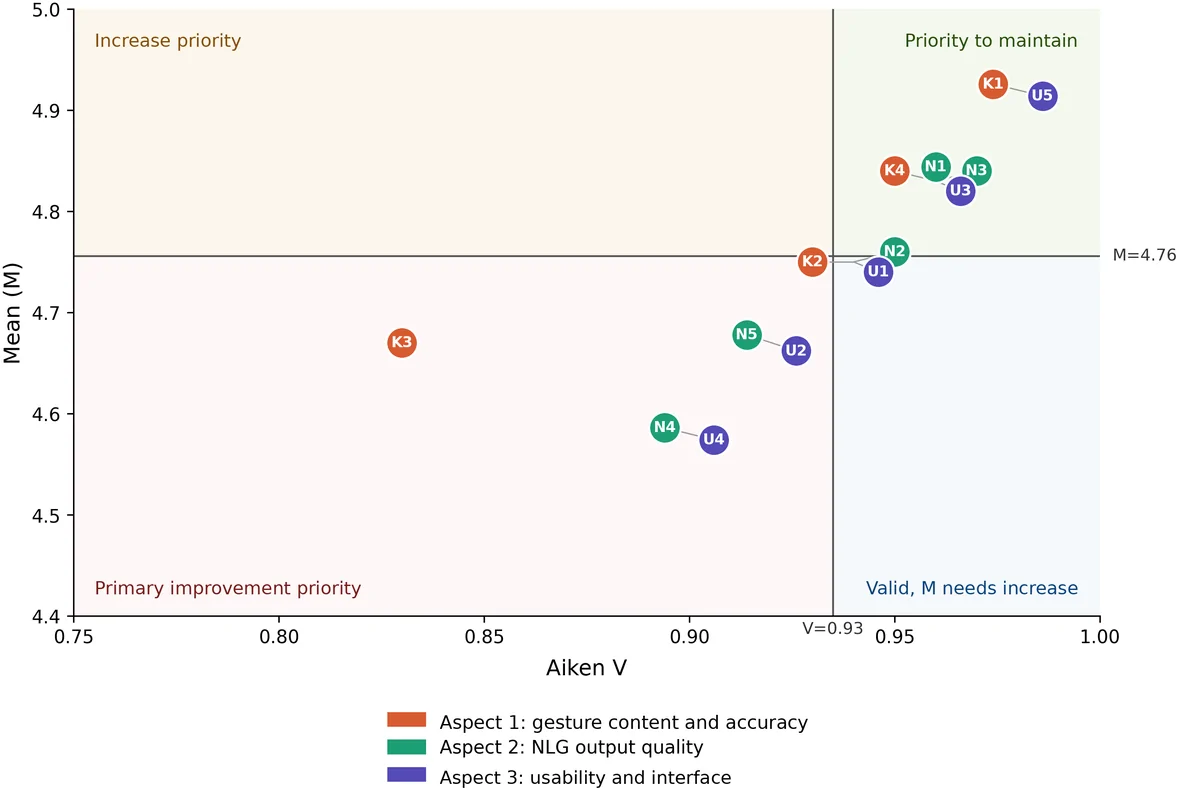
Cartesian diagram of expert validation results. NLG: natural language generation.

The Cartesian diagram of Figure 5 shows the 2 indices plotted simultaneously against the 14 validation items. The line is drawn at this average Aiken V of 0.94 (SD 0.04) with the average of 4.76 (SD 0.11). In the first quadrant in the top right (priority to maintain) are K1, K4, N1, N3, U3, and U5. The base strengths of the prototype are indicated by a V value greater than 0.94 and a mean value greater than 4.76 (SD 0.11). K3, N4, and U4 are located in the lower left quadrant. The 3 are interrelated systematically, and these 3 items would constitute the targeted area of improvement in the next round.

### Field Demonstration and Evaluation

Field testing was conducted using 3 complementary instruments across 2 Indonesian cities (Table 4).

**Table 4.** Field evaluation results (Makassar and Malang)^a^.

Respondent	Makassar, mean (SD); n/N (%)	Malang, mean (SD); n/N (%)	Combined, mean (SD); n/N (%)
User participants	3.34 (0.16); 8/15 (53)	3.36 (0.17); 7/15 (47)	3.35 (0.16); 15/15 (100)
Observers/companions	4.35 (0.07); 2/4 (50)	4.45 (0.07); 2/4 (50)	4.40 (0.08); 4/4 (100)

a Mean scores are on a 5-point Likert scale. SD is the sample SD (n−1) of respondent-level mean scores. Mean score as a percentage of the maximum score (5): user participants, 67% (Makassar), 67% (Malang), 67% (combined); observers/companions, 87% (Makassar), 89% (Malang), 88% (combined).

In both Makassar and Malang, ratings from observers and companions were consistently higher than ratings from user participants. The reported practicality percentages represent mean Likert-scale ratings converted to percentages of the maximum possible score, rather than proportions of participants. For overall practicality, user participants with communication disorders gave an average score of 3.35 (SD 0.16) out of 5, or 67% (n=15). Conversely, observers and companions gave an average score of 4.40 (SD 0.08), or 88% (n=4). The gap in scores was as high as about 24 percentage points in Makassar and 18 percentage points in Malang. In Surabaya, an independent disability specialist rated the utility of the prototype at 81% (with a mean value calculated across the 20 questionnaire items of 4.05, SD 0.60; n=1), which backs up the higher scores from observers and companions. The Makassar (only user) responses revealed the lowest-scoring aspects of voice output suitability and clarity. Both sites repeatedly mentioned the gloves’ sensitivity, that they are easy to use but not intuitive, the regional variation in BISINDO terminology, and that user guides would need to be produced.

Our comparison involves linguistic analysis of the unprocessed raw input created by gestures (the system input) and the sentence generated by AI (the system output) for each interactive simulation. Three transformation processes were identified. Addition was used to combine elements for a fuller answer, that is, “I” + “eat” became “I am eating.” The process of elision removed redundant elements, for example, “I” + “already” + “drink” became “I drink water.” Finally, “air” was introduced and “already” omitted, which shows how an output can go through multiple operations at the same time. Substitution occurred when the elements were replaced, causing the meaning to change, for example, “mother” + “running” became mother walking, not mother running. Among the 3 processes, substitution was the worst, because it not only introduced grammar changes but also changed what the user intended to say. Thus, this is consistent with the relatively lower user ratings of the speech output with respect to appropriateness and clarity. Any replacement effectively changes the meaning of the original sentence, causing it to become no longer what the speaker intended. Regional differences in BISINDO vocabulary were also found across sites, indicating that additional training data of the same target words from different regions is necessary.

## Discussion

### Principal Results

Four principal findings emerged from this study. First, cross-disciplinary stakeholder consensus, assessed quantitatively through the needs analysis, confirmed a very high and consistent priority for BISINDO-based assistive communication technology capable of generating natural, real-time speech (grand mean 4.77, SD 0.51; mean agreement 89%). Second, the SmartGlove BISINDO prototype directly addresses this need through a 5-layer architecture that, to our knowledge, is the first BISINDO system to integrate LLM-based NLG into the translation pipeline. Third, expert validation indicated strong content validity (grand mean Aiken V 0.94, SD 0.04; CVR 0.76, SD 0.16; 11 of 14 items highly valid), providing preliminary evidence that the prototype design is ready to proceed to the next DSR cycle. Fourth, field demonstration with actual users with communication disorders showed favorable practicality ratings from observers, companions, and a disability expert (81%‐88%), but lower ratings from user participants themselves (67%), revealing a gap between expert-perceived feasibility and lived user experience that motivates further design refinement.

From a linguistic perspective, items assessing the grammaticality and naturalness of AI-generated sentences (N1, V=0.96) received highly valid ratings, suggesting that the AI Language Wrapper output meets the ideational and textual metafunctions defined by SFL. Perfect agreement on interface inclusivity (U5, V=0.98) similarly supports the AAC principle of independent operability. Field-based linguistic analysis supports the expert’s validity ratings. This analysis identified substitution, rather than addition or deletion, as the transformation process most likely to disrupt ideational metafunctions. This is because substitution alters the intended meaning (field demonstration and evaluation, and results).

In this study, the design of the BISINDO dataset was conducted in collaboration with certified BISINDO experts. This meant prioritizing vocabulary that people actually use in conversation while eliminating classes that are rarely encountered or difficult to visually distinguish. This stakeholder-driven refinement aligns with user-centered design principles, which are crucial for achieving adoption of rehabilitation technology in real-world practice settings [37]. Co-design with experts is a key implementation driver that requires ongoing effort.

These findings carry implications at 3 levels. At the practice level, SmartGlove BISINDO has the potential to reduce communication barriers for individuals with speech-impairment in Indonesian inclusive education classrooms, public service interactions, and daily social contexts, settings in which no deployable BISINDO-to-speech solution currently exists. Given that an estimated 1.02% of Indonesia’s population experiences severe communication difficulties [4], even modest adoption of this prototype could yield meaningful benefits for public health and educational equity, supporting SDG 4 and SDG 10. At the methodological level, the DSR-grounded, co-design-intensive development process demonstrated here offers a preliminary, potentially transferable model for AT research in other underrepresented sign language communities, particularly those facing similar constraints in dataset availability and NLG integration; this transferability remains to be empirically tested rather than assumed. At the policy level, the strong stakeholder consensus on authentic BISINDO vocabulary and independent operability provides preliminary evidence to inform advocacy for context-sensitive, locally grounded assistive communication solutions, consistent with calls to address structural barriers to technology adoption in low- and middle-income countries [38].

### Comparison With Prior Work

Table 5 situates SmartGlove BISINDO against previous BISINDO recognition systems, and the most recently published related work is shown in Table 5.

**Table 5.** Comparison of SmartGlove Bahasa Isyarat Indonesia (BISINDO) with prior BISINDO recognition systems.

Study	Input modality	Recognition level	NLG^a^/sentence generation	Reported accuracy, %
Aljabar and Suharjito [10], 2020	Camera (CNN-LSTM)^b^	Isolated gesture	No	96
Candra et al [11], 2024	Camera (CNN-MobileNetV2, mobile)	Isolated gesture (30 classes)	No	55
Wiguna and Rojali [12], 2025	Camera (3D CNN-LSTM/BiLSTM^c^+CTC^d^)	Sentence-level (gesture-to-text only)	No	Not directly comparable
Kindy et al [14], 2024	Camera (Transformer: SPOTER^e^, Siformer, MViTv2^f^)	Isolated sign, word-level	No	97
Fadzli and Rahardi [15], 2026	Camera (MobileNetV3, EfficientNetV2-B0)	Isolated hand sign	No	90‐96
Maharjan et al [39], 2025	Wearable glove (hybrid CNN-Transformer)	Isolated gesture	No	98
SmartGlove BISINDO (this study)	Wearable dual-glove (flex+IMU^g^ sensors)	Isolated gesture, LLM^h^-corrected to full sentence	Yes (LLM-based NLG)	Target ≥85%; empirical validation pending

a NLG: natural language generation.

b CNN-LSTM: convolutional neural network–long short-term memory.

c CNN-BiLSTM: convolutional neural network–bidirectional long short-term memory.

d CTC: connectionist temporal classification.

e SPOTER: Sign Pose-based Transformer.

f MViTv2: Multiscale Vision Transformer, version 2.

g IMU: inertial measurement unit.

h LLM: large language model.

A closely related system for Indonesian Sign Language, though developed for SIBI rather than BISINDO, integrated an LSTM gesture classifier with an IndoBERT language model and reported 89% gesture accuracy alongside a 3-fold improvement in word-prediction accuracy when the language model was applied [13]. This confirms that combining gesture recognition with language modeling improves output quality even in closely related Indonesian sign systems, though this approach remained limited to word-level correction rather than full sentence generation. Among wearable glove-based systems, a category of approaches reviewed extensively in the broader wearable sensor-based sign language recognition literature [40], Maharjan et al [39] reported 98% gesture classification accuracy using a hybrid CNN-Transformer model, though this system, like other prior work, does not incorporate NLG for sentence-level output.

Of all systems compared, only SmartGlove BISINDO stands apart. It’s the only one that combines wearable sensors with LLM-based NLG to produce full sentences based not on words/gesture-to-text, but on other gestures. Remember, this comparison used a narrative synthesis of previous results rather than a direct head-to-head trial. This is not a fair apples-to-apples comparison since the systems are different in data sets, hardware, and evaluation protocols. This limitation is expanded upon in the Limitations section below. Additionally, it was not used to measure the gesture-recognition accuracy of SmartGlove BISINDO itself in this study. Instead, evaluation looked at content validity through an expert panel and field practicality with users, observers, and a disability expert. Technical classification performance wasn’t the focus, and that’s discussed under Future Directions.

### Limitations

Consistent with common methodological challenges in evaluating rehabilitation technologies [30], field evaluation was limited to practicality ratings from a small sample (15 user participants, 4 observers/companions, 1 disability expert) across 2 to 3 cities, rather than a controlled effectiveness trial with a comparison condition or pre-post outcome measures; results should therefore be interpreted as an early-stage field demonstration (DSR phases 4‐5) rather than a definitive effectiveness evaluation. Similarly, the 12-participant expert panel, while meeting the minimum threshold for CVR and Aiken V analyses [31,32], limits the generalizability of the needs-analysis and validation findings, and a formal interrater reliability coefficient was not computed given the sample size. Because both instruments were developed specifically for this study, empirical construct validation beyond expert content review, such as confirmatory factor analysis with a larger sample, was not conducted.

Several sources of uncertainty in real-time gesture recognition were not directly quantified, including sensor noise, classification ambiguity near the 85% or higher confidence threshold, and intersigner variability in gesture speed and amplitude; because the AI Language Wrapper generates sentences from already-classified gestures, such uncertainty can propagate into the output, for example, a misclassified gesture with falsely high confidence could yield a sentence that is grammatically fluent but semantically incorrect. Quantifying this uncertainty, for example through confidence-aware generation or user-facing indicators, is an important direction. Additional hardware limitations include the need for more ergonomic glove materials and sensor straps to support longer sessions, and the fact that the target TFLite model accuracy of ≥85% across diverse user cohorts and all 150 gesture classes has not yet been empirically validated.

### Future Directions

Building on the preliminary evidence for content validity established here, 4 directions define the research agenda going forward. First, the gap between favorable observer/expert ratings and lower user-participant ratings, particularly for speech-output clarity, warrants deeper investigation; we recommend incorporating the technology acceptance model [41] alongside AAC and SFL as an interpretive framework, since the technology acceptance model’s core constructs of perceived usefulness and ease of use are well suited to explaining why expert-perceived feasibility diverges from lived user experience. Second, empirical performance testing is needed to verify the TFLite gesture-classification model’s accuracy across diverse user cohorts and hand morphologies and to quantify the classification and language-generation uncertainties identified in the Limitations section under real-world conditions. Third, the priority-improvement areas identified through expert validation point to specific refinements for the next DSR design cycle: similar-gesture differentiation (K3) toward sensor augmentation or finer feature engineering, contextual relevance of AI-generated output (N4) toward more sophisticated, BISINDO-specific prompt engineering, and settings configuration ease (U4) toward interface simplification. Fourth, scalability testing to other underrepresented sign language systems in Southeast Asia is planned, building on the language-agnostic design of Layers 1 to 3; given that over 50% of individuals in some low- and middle-income countries lack access to needed rehabilitation services [42], portable, low-infrastructure solutions such as SmartGlove BISINDO carry urgency beyond Indonesia, though this broader applicability remains to be empirically demonstrated rather than assumed.

### Conclusions

This study developed and validated SmartGlove BISINDO, which is, to our knowledge, the first BISINDO sign language–to-speech system to integrate LLM-based NLG, addressing a systemic gap in prior BISINDO recognition research. A needs analysis of 12 cross-disciplinary participants confirmed a very high and consistent priority (grand mean 4.77, SD 0.51, mean agreement 89%) across all 4 need clusters: response speed and accuracy, naturalness of language output, portability and ease of use, and cultural and linguistic relevance.

The resulting prototype comprises a 5-layer architecture spanning dual-sensor glove hardware, wireless communication, on-device gesture classification, LLM-based sentence generation, and speech output. Expert validation by the same 12 participants indicated strong content validity (grand mean Aiken V 0.94 (SD 0.04), CVR 0.76 (SD 0.16), both exceeding their respective thresholds), with 11 of 14 items rated highly valid. Three items, concerning similar-gesture differentiation, contextual relevance of generated output, and settings configuration ease, were identified as priorities for refinement in the next DSR design cycle iteration. Field demonstration with actual users, observers, and a disability expert further indicated favorable practicality overall, alongside a gap between expert-perceived feasibility and user-rated experience that will guide the next design iteration.

## Supplementary material

10.2196/96258 Multimedia Appendix 1 Clusters and items of the needs analysis questionnaire.

10.2196/96258 Multimedia Appendix 2 Aspects and items of the expert validation sheet.

10.2196/96258 Multimedia Appendix 3 SmartGlove BISINDO dataset composition by category.

## Abbreviations

AAC: augmentative and alternative communication
AT: assistive technology
BERT: Bidirectional Encoder Representations from Transformers
BISINDO: Bahasa Isyarat Indonesia
BLE: Bluetooth Low Energy
BLEU: Bilingual Evaluation Understudy
CNN: convolutional neural network
CVR: content validity ratio
DSR: design science research
IMU: inertial measurement unit
LLM: large language model
LSTM: long short-term memory
MViTv2: Multiscale Vision Transformer version 2
NLG: natural language generation
REST: Representational State Transfer
SDG: Sustainable Development Goal
SFL: systemic functional linguistics
SIBI: Indonesian Sign System
SPOTER: Sign Pose-based Transformer
TTS: text-to-speech
