# Supplementary material for: The SmartGlove BISINDO Wearable Sensor and Large Language Model–Based Sign Language–to-Speech System for Users With Communication Disorders: Design Science Research Study
Source: JMIR Rehabil Assist Technol. 2026 Oct 22;13:e96258. doi: 10.2196/96258 (PMC13690336; doi:10.2196/96258)
Supplement: Supplementary file 1 [file rehab-v13-e96258-s001.docx]

**Multimedia Appendix 1. Clusters and items of the needs analysis questionnaire.**

**Table.** Clusters and Items of the Needs Analysis Questionnaire

| **No** | **Cluster** | **Items** | **Indicators** |
| --- | --- | --- | --- |
| 1 | Response Speed & Accuracy | 3 | Speech output <1 second; real-time gesture recognition; minimizing false detection errors |
| 2 | Naturalness of Language Output | 3 | Complete grammatical sentences; comprehensible to lay conversation partners; formal/informal context adjustment |
| 3 | Portability & Ease of Use | 2 | Lightweight portable device; independent operation without assistance |
| 4 | Cultural & Linguistic Relevance | 2 | Based on authentic BISINDO vocabulary, coverage of at least 150 gestures |
|  | **Total** | **10** |  |
