# Supplementary material for: The SmartGlove BISINDO Wearable Sensor and Large Language Model–Based Sign Language–to-Speech System for Users With Communication Disorders: Design Science Research Study
Source: JMIR Rehabil Assist Technol. 2026 Oct 22;13:e96258. doi: 10.2196/96258 (PMC13690336; doi:10.2196/96258)
Supplement: Supplementary file 2 [file rehab-v13-e96258-s002.docx]

**Multimedia Appendix 2. Aspects and items of the expert validation sheet.**

**Table** Aspects and Items of the Expert Validation Sheet

| **No** | **Aspect** | **Items** | **Coverage** |
| --- | --- | --- | --- |
| 1 | BISINDO Gesture Content & Accuracy | 4 | Vocabulary authenticity, gesture class coverage, similar-gesture differentiation, sensor accuracy |
| 2 | NLG Output Quality & Sentence Naturalness | 5 | Grammaticality, naturalness, word order correction, contextual relevance, and AI response latency |
| 3 | Usability & Application Interface | 5 | Ease of operation, status indicators, conversation history, settings configuration, and inclusive design |
|  | **Total** | **14** |  |
