# Supplementary material for: The SmartGlove BISINDO Wearable Sensor and Large Language Model–Based Sign Language–to-Speech System for Users With Communication Disorders: Design Science Research Study
Source: JMIR Rehabil Assist Technol. 2026 Oct 22;13:e96258. doi: 10.2196/96258 (PMC13690336; doi:10.2196/96258)
Supplement: Supplementary file 3 [file rehab-v13-e96258-s003.docx]

**Multimedia Appendix 3. SmartGlove BISINDO dataset composition by category.**

**Table** SmartGlove BISINDO Dataset Composition

| **No** | **Category** | **Classes** | **Coverage** | **Status** |
| --- | --- | --- | --- | --- |
| 1 | Letters (A–Z) | 26 | All 26 BISINDO alphabet letters | Complete |
| 2 | Numbers | 42 | 1–30, tens, hundreds, thousands, millions, billions | Complete |
| 3 | Greetings & Social | 21 | Greetings, introductions, morning/afternoon/evening/night | Complete |
| 4 | Family | 18 | Nuclear and extended family (uncle, aunt, cousin, niece/nephew) | Complete |
| 5 | Core Essential Words | 2 | Understand, help | Complete |
| 6 | Important Additions | 41 | Pronouns, verbs, adjectives, emergency phrases | ~80% |
| **Total** | | **150** |  | **±120/150 (±80%)** |
